# Automatic inference of hypoglycemia causes in type 1 diabetes: a feasibility study

**DOI:** 10.3389/fcdhc.2023.1095859

**Published:** 2023-04-17

**Authors:** Aleksandr Zaitcev, Mohammad R. Eissa, Zheng Hui, Tim Good, Jackie Elliott, Mohammed Benaissa

**Affiliations:** ^1^ Department of Electronic and Electrical Engineering, University of Sheffield, Sheffield, United Kingdom; ^2^ Department of Oncology and Metabolism, University of Sheffield, Sheffield, United Kingdom; ^3^ Department of Diabetes and Endocrinology, Sheffield Teaching Hospitals NHS FT, Sheffield, United Kingdom

**Keywords:** biomedical informatics, classification algorithms, machine learning, medical expert systems, statistical analysis, hypoglycemia, exercise, physical activity

## Abstract

**Background:**

Hypoglycemia is the most common adverse consequence of treating diabetes, and is often due to suboptimal patient self-care. Behavioral interventions by health professionals and self-care education helps avoid recurrent hypoglycemic episodes by targeting problematic patient behaviors. This relies on time-consuming investigation of reasons behind the observed episodes, which involves manual interpretation of personal diabetes diaries and communication with patients. Therefore, there is a clear motivation to automate this process using a supervised machine learning paradigm. This manuscript presents a feasibility study of automatic identification of hypoglycemia causes.

**Methods:**

Reasons for 1885 hypoglycemia events were labeled by 54 participants with type 1 diabetes over a 21 months period. A broad range of possible predictors were extracted describing a hypoglycemic episode and the subject’s general self-care from participants’ routinely collected data on the Glucollector, their diabetes management platform. Thereafter, the possible hypoglycemia reasons were categorized for two major analysis sections - statistical analysis of relationships between the data features of self-care and hypoglycemia reasons, and classification analysis investigating the design of an automated system to determine the reason for hypoglycemia.

**Results:**

Physical activity contributed to 45% of hypoglycemia reasons on the real world collected data. The statistical analysis provided a number of interpretable predictors of different hypoglycemia reasons based on self-care behaviors. The classification analysis showed the performance of a reasoning system in practical settings with different objectives under F1-score, recall and precision metrics.

**Conclusion:**

The data acquisition characterized the incidence distribution of the various hypoglycemia reasons. The analyses highlighted many interpretable predictors of the various hypoglycemia types. Also, the feasibility study presented a number of concerns valuable in the design of the decision support system for automatic hypoglycemia reason classification. Therefore, automating the identification of the causes of hypoglycemia may help objectively to target behavioral and therapeutic changes in patients' care.

## 1 Introduction

The general goal of type 1 diabetes (T1D) care is maintenance of near normal levels of blood glucose (BG) (1). Avoidance of higher than normal BG (hyperglycemia) reduces the risk of long-term complications, such as eye, kidney or nerve damage (2). Whilst avoidance of low BGs (hypoglycemia) reduces the risk of cognitive impairment and accidents. Successful T1D management largely depends on patient self-care, which requires education and frequent decision making including regular blood glucose (BG) monitoring, insulin intake and its dose adjustment, carbohydrate counting, and planning for dose adjustments due to physical activity (3).

**Figure 1:**
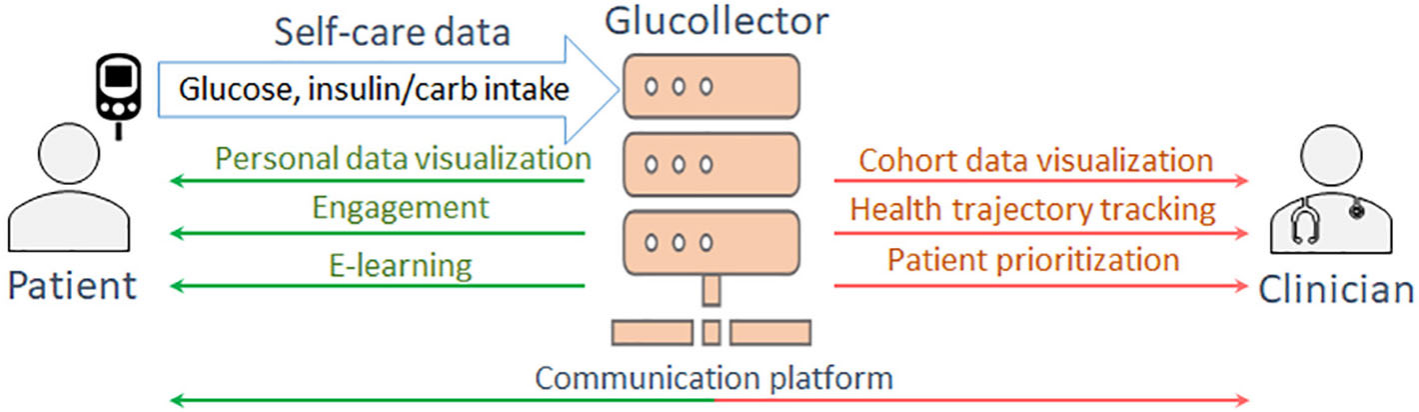
The functionality of Glucollector.

In conventional diabetes care BG levels and food/medication intake are registered by patients in individual diabetes diaries and are examined by clinicians at personal appointments generally occurring once every 3-6 months (4). However, with current and projected advances in sensing and communication technologies, diabetes care is progressively becoming more data-driven and patient-centered, focused on e-learning, decision support and remote supervision of self-care practices based on multimodal data acquisition (5–9). A novel developed Glucollector system (**Figure 1**) follows this approach. This data acquisition and management suite facilitates the collection, management and visualization of self-monitored blood glucose (SMBG) measurements, continuous glucose monitoring (CGM) data, carbohydrate and insulin intake, and provides a platform for clinician-patient communication and e-learning. Glucollector has been deployed in the DAFNEplus (Dose Adjustment for Normal Eating) Randomized Controlled Trial (RCT) (10).

In both personal appointments and remote monitoring, the clinicians supervisory role is to detect problematic self-care patterns in diabetes diaries, to clarify the context of the abnormal pattern with the patient to identify the underlying reason, and to suggest changes to self-care routines to avoid such patterns reoccurring. Such manual interpretation of every episode is problematic considering the time lags between personal appointments and the amounts of data aggregated between them. This process is more feasible in a day-to-day patient supervision paradigm facilitated by the diabetes data management system, such as the Glucollector, where clinicians can examine the remotely collected self-care data in a convenient form. However, this process still becomes a tedious routine for clinicians often supervising tens or hundreds of patients, and therefore, there is a clear motivation to automate such BG pattern interpretation and provide clinical decision support driven by artificial intelligence (AI) and machine learning technology.

The modern diabetes treatment is associated with large amounts of data generated from health records, food/medication diaries and various smart monitoring devices. Taking this into account and that hypoglycemia represents a major adverse consequence of diabetes treatment, substantial research effort was committed to design data-driven tools based on machine learning that would provide assistance in clinical practice and personal day-to-day hypoglycemia prevention. A number of recent systematic reviews outline these research efforts (11–14). However, from these studies it is evident that in context of hypoglycemia prevention the pattern recognition and machine learning research mainly focuses on hypoglycemic event prediction, while the retrospective data-driven interpretation of reasons behind such events (automated hypoglycemia cause classification), that is needed for behavioral intervention, remains understudied.

This manuscript aims to fill this gap by conducting a feasibility study aiming to determine the practicality and limitations of automated hypoglycemia reason classification from patterns of T1D self-care data, collected by the modern diabetes management software. The key objectives of this study are to design indicative predictor variables from time series of self-care data, assess their statistical relationship with reasons behind hypoglycemic events, design an automated system for hypoglycemia cause classification and assess its practicality.

## 2 Materials and methods

### 2.1 Data acquisition

Data was collected between March 2018 and December 2019 from a cohort of 54 Glucollector users who were also patients of Sheffield Teaching Hospitals (STH) NHS Foundation Trust taking part in the DAFNEplus RCT, for details refer to the protocol paper of the trial (10). Ethics approvals were granted under REC ref: 16/NW/0573 and 18/SW/0100. For the purpose of data collection, the 10 most common reasons behind hypoglycemia episodes, defined as < 4mmol/L SMBG measurements, were identified by the diabetes specialists based on their clinical practice and patient interviews.

These reasons were presented to the participating people with type 1 diabetes who were asked to label their recent hypoglycemia episodes from the preceding two weeks on their Glucollector diabetes diary interface. For every episode, they were allowed to select up to two possible reasons with a confidence level of 1 to 5 for every answer with 5 being the highest confidence.

**Figure 2:**
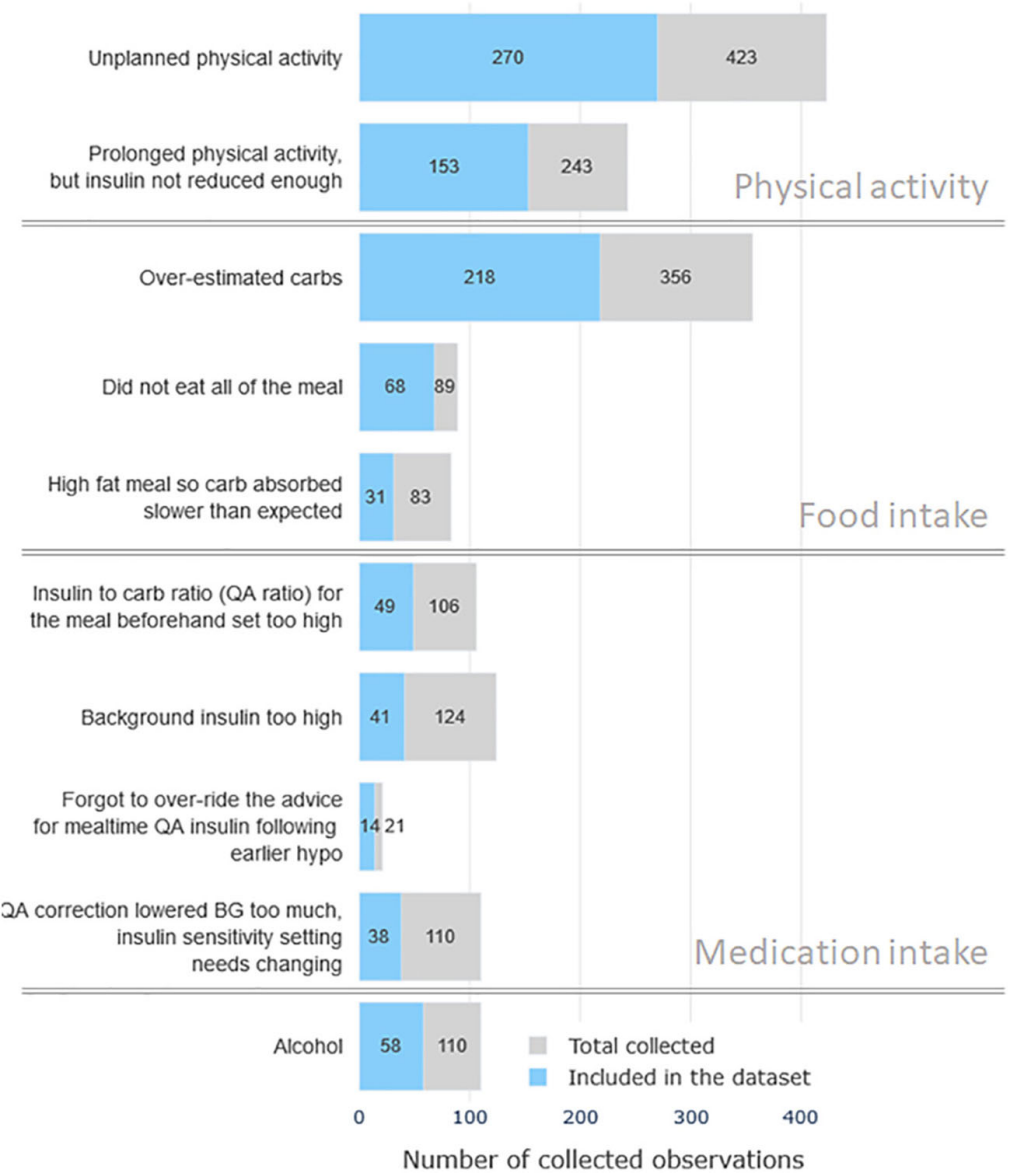
The distribution of the observed hypoglycemia cases by reasons. Gray color bars mark the total number of collected cases and the blue color bars denote the number of high-confidence cases included in the analysis dataset.

The 54 participants were 36 female (18 male), and the average age was 46.8± 15.2 years. These participants labeled a total of 1885 hypoglycemia observations which were obtained during the data collection period. Episodes that did not belong to class “Other” or “Don’t know” and which were labeled with confidence levels 4-5 were included into the analysis dataset, yielding a total of 821 observations. **Figure 2** shows the class distribution of the collected dataset. Considering the vast disbalance in class observations the cases were grouped into the three general categories of hypoglycemia causes for statistical and predictive analysis: physical activity, mistakes in food intake, mistakes in medication dosage selection. It can be noted that cases related to physical activity constitute about 45% of the dataset. The second most prominent category of episodes is associated with carbohydrate counting and food intake (≈33%). Cases related to insulin dosage selection and intake comprise the third most common category with ≈15%. These three groups are used as possible class labels in the classification analysis section, i.e. the proposed machine learning system aims to “guess” the cause of hypoglycemic event from patterns of self-care data around the event.

### 2.2 Event representation

For purposes of statistical and classification analyses discussed in the further sections, each labeled hypoglycemic case was represented as a set of predictor variables or “features” that describe the context of the observed hypoglycemia. These features were extracted from a variety of data sources in the Glucollector: static subject demographics from health records, user-uploaded self-care data (BG, insulin intake, carbohydrate intake), and Glucollector website usage statistics.

One of the key stages of the proposed analysis is to identify factors in data that are indicative of a particular hypoglycemia episode. To the best of our knowledge no such analyses have been conducted before, therefore, a broad range of possible predictors describing both the episode and the subject’s general self-care were considered. **Table 1** lists the extracted features by categories, gives their description and short encodings. Each hypoglycemic case was represented by a total of 83numerical variables. Features that are not self-explanatory are further detailed below.

**Table 1:** Considered predictor variables.

#	Feature description	Encoding	#	Feature description	Encoding
	**Demographics**		40	Total insulin advice	ba_total_ins
1	Age	age	41	Difference between advised and logged intake	ba_diff
2	Gender	gender	42	Meal rise setting	ba_meal_rise
3	Years with diabetes	y_w_diab	43	The presence of ‘Excercise’ tag before the event	ba_has_excercise
	**Self-care during the episode**			**General self-care behavior over the past 3 months**	
	**Temporal features**			**Blood glucose**	
4	Relative time of day as a single float variable [0,1]	tod	44	BG mean	glu_mean
5	Absolute time offset from noon	cos_tod	45	BG standard deviation	glu_std
6	Absolute time offset from midnight	sin_tod	46	BG range	glu_range
7	Local offset from the usual wake up time	wake_up_offset	47	BG maximum	glu_max
8	Is business day	is_bday	48	BG minimum	glu_min
	**Interpolated BG curve**		49	BG rate of mean value crossing	glu_mean_x
8	Mean of interpolated BG	bgi_mean	50	BG skewness	glu_skew
9	Standard deviation of interpolated BG	bgi_std	51	BG kurtosis	glu_kurt
10	Range of interpolated BG	bgi_range	52	Mean of negative BG slopes	neg_slopes_mean
11	Minimum of interpolated	BG_bgi_min	53	Mean of positive BG slopes	pos_slopes_mean
12	Maximum of interpolated BG	bgi_max		**QA insulin intake dosages**	
13	Slope of interpolated BG	bgi_slope	54	QA insulin mean	ins_mean
14	Skewness of interpolated BG	bgi_skew	55	QA insulin standard deviation	ins_std
15	Kurtosis of interpolated BG	bgi_kurt	56	QA insulin range	ins_range
	**Recent SMBG before the episode**		57	QA insulin maximum	ins_max
16	Previous BG reading value	pre_g_val	58	QA insulin minimum	ins_min
17	Time offset from the previous BG reading	pre_g_offset	59	QA insulin rate of mean value crossing	ins_mean_x
18	Time offset from the previous hyperglycemia	pre_H_offset	60	QA insulin skewness	ins_skew
19	Time offset from the previous hypoglycemia	pre_h_offset	61	QA insulin kurtosis	ins_kurt
20	Time offset from the previous in-target reading	pre_t_offset		**Basal insulin intake dosages**	
	**SMBG after the episode**		62	Basal insulin mean	bas_mean
21	Next BG reading value	post_g_val	63	Basal insulin standard deviation	bas_std
22	Time offset to the next BG reading	post_g_offset	64	Basal insulin range	bas_range
23	Time offset to the next hyperglycemia	post_H_offset	65	Basal insulin maximum	bas_max
24	Time offset to the next hypoglycemia	post_h_offset	66	Basal insulin minimum	bas_min
25	Time offset to the next in-target reading	post_t_offset	67	Basal insulin rate of mean value crossing	bas_mean_x
	**QA insulin intake**		68	Basal insulin skewness	bas_ skew
26	Previous QA insulin dosage	pre_ins_dosage	69	Basal insulin kurtosis	bas_kurt
27	Time offset from previous QA insulin intake	pre_ins_offset		**Carbohydrate intake**	
28	Next QA insulin dosage post ins dosage		70	Carb mean	car_mean
29	Time offset to the next QA insulin intake	post_ins_offset	71	Carb standard deviation	car_std
	**Basal insulin intake**		72	Carb range	car_range
30	Previous basal insulin dosage	pre_bas_dosage	73	Carb maximum	car_max
31	Time offset from previous basal insulin intake	pre_bas_offset	74	Carb minimum	car_min
32	Next basal insulin dosage	post_bas_dosage	75	Carb rate of mean value crossing	car_mean_x
33	Time offset to the next basal insulin intake	post_bas_offset	76	Carb skewness	car_skew
	**Food intake**		77	Carb kurtosis	car_kurt
34	Previous carbohydrate intake amount	pre_car_dosage		**Engagement with GlucoChallenge quiz**	
35	Time offset from previous carbohydrate intake	pre_car_offset	78	Number of quiz answers	cnc_ansnum
36	Next carbohydrate intake amount	post_car_dosage	79	Number of separate days with any quiz answers	cnc_daysnum
37	Time offset to the next carbohydrate intake	post_car_offset	80	Average of quiz points	cnc_points_avg
	**Bolus advisor**		81	Standard deviation of quiz points	cnc_points_std
38	Most recent food insulin advice	ba_food_ins	82	Average number of answers per session	cnc_dist_avg
39	Most recent correction insulin advice	ba_corr_ins	83	Standard deviation of answers per session	cnc_dist_std

The temporal features section contains the absolute time offsets in hours from noon and midnight to alleviate the boundary issue of a single float time representation (tod feature), e.g. 23:55 and 00:05 being represented by a very large and a very small value respectively, while being just 10 minutes apart. The *wake_up_offset* feature is extracted as a difference between the 3 month median time of day’s first BG reading (after 4:30) and the episode day’s first BG reading time. This feature was included to describe the discrepancy from subject’s usual day schedule. All time offsets (features 5-7, 17-20, 22-25, 27, 29, 31, 33, 35, 37) were expressed in hours.

Features 8-15 in **Table 1** encode the BG trajectory around the hypoglycemic episode. To obtain them, SMBG readings from the 6 hours long time segment around each episode were interpolated using the piecewise cubic Hermite interpolating polynomial (PCHIP) (15) and summarized using a number of commonly used statistical functionals.

Features 26, 28, 30, 32, 34, 36 describing the most recent and the next intakes of food/medication were centered using the subject-specific means in order to take into account subjects’ physiological differences and their general food/medication intake amounts. For example, with $I_{mean\_k}$ being the average QA (quick acting) insulin dosage for subject k (feature 54), and $I_{pre\_k}$ being their most recent QA insulin intake dosage in a particular episode, the feature 26 was expressed as a difference $I_{pre\_k}-I_{mean\_k}$ .

Features 38-43 were extracted from patient personal Bolus Advisor devices which were used for QA insulin dosage calculation shortly before episodes.

The general self-care features 44-83 describe the overall statistics of BG, food and medication intake over the 3 months preceding the episode. In addition to that, each case included features of engagement with one of the Glucollector’s e-learning tools GlucoChallenge, which reflects the subject ability to estimate carbohydrate amount.

### 2.3 Statistical analysis

The aim of this analysis is to characterize the patterns of self-care during the different categories of causes of hypoglycemia. More specifically, the aim is to identify statistically significant relationships between the considered predictors and the target class labels. This analysis was conducted by fitting statistical models with binary outputs separately for each considered class of hypoglycemia.

The dataset is characterized by the three important qualities, which define the applicable types of such analysis: the observations are clustered by subjects, the considered predictors have different distributions and a high degree of collinearity. The former arises from the fact that each subject has provided multiple observations, which violates the observation independence assumption of the majority of statistical modeling tools. Therefore, Mixed Effect Generalized Linear Models (MEGLM) (16) and Generalized Estimating Equations (17) were considered, as they are capable of dealing with panel data and have no assumptions about the variable distributions.

**Figure 3:**
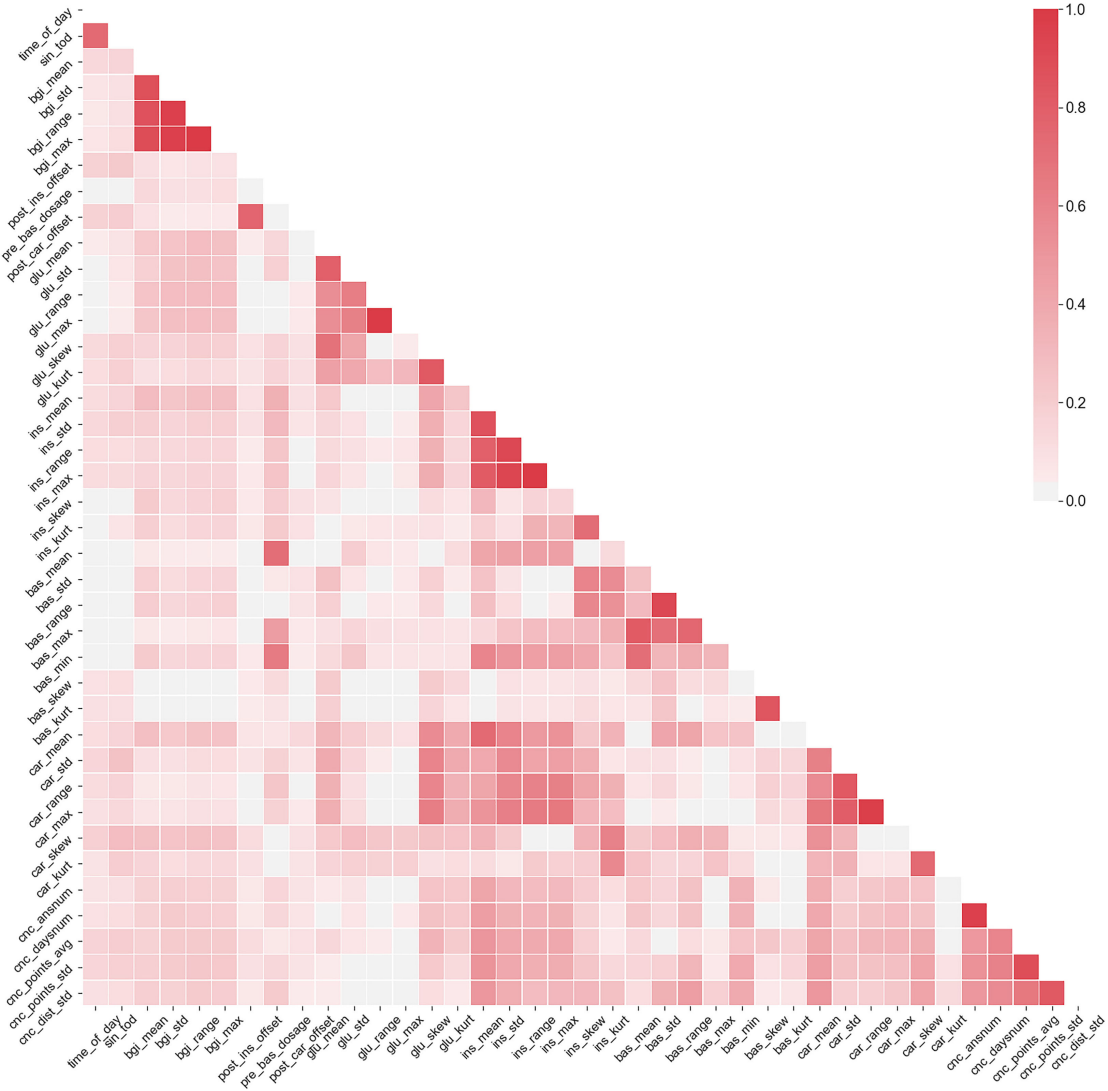
The correlation matrix of features that have exhibited *ρ*≥0.7 with at least one other feature. Color scale denotes the correlation between the variables.

The multicollinearity means that many of the considered data features are correlated. While there are no formal orthogonality assumptions in MEGLM and GEE methods, statistical significance of effect size estimates and model stability are deteriorated in the presence of highly collinear features (18). In the selected dataset the collinearity is mainly intrinsic and arises from how the predictors are defined, for example the predictor insulin range naturally correlates with both predictors minimum insulin value and maximum insulin value. **Figure 3** shows the correlation matrix of the highly collinear non-binary features (Pearson *ρ*≥ 0.7 with any other feature).

The statistical significance of the relationships between the considered predictors and the target class labels were analyzed by fitting statistical models with binary outputs for each considered class of hypoglycemia. Not all of the considered features were available in some of the episode observations, especially the ones related to Bolus Advisor settings as they were often not provided by the users. This significantly reduces the dataset size in multiple regression analysis. Therefore, two types of regression were employed - single variable regression, allowing for a larger sample size, and multiple regression, allowing for a better model fit.

MEGLM models with binary output, logit link and random subject-specific intercepts were used in both single-variable and multiple regressions. This method was chosen over GEE due to the improved convergence stability. The generalized form of a MEGLM model is defined as follows:


(1) g(μ)=Xβ+Zb+δ 

where *g* is the link function, *μ* is n-by-1 vector of conditional probability of the outcome given the random effect *b* with n denoting the number of observations (events). ***X*** is n-by-f design matrix of predictor variables from **Table 1** with f denoting the number of features. *β* is the f-by-1 fixed effects vector. ***Z*** is n-by-q random effect design matrix and b is the random effects q-by-1 vector with q denoting the number of participants. *δ* is the model offset vector.

With logit link function, subject id *i*, observation (event) *j*, hypoglycemia category *k*, single predictor variable *x* from **Table 1**, equation (1) takes the following form:


(2) $$logit\left(p_{ijk}\right)=ln\left(\frac{p_{ijk}}{1-p_{ijk}}\right)=\beta_{0}+\beta_{1}x_{ij}+b_{i}+\epsilon_{ijk}\;$$

where *p_ijk_* is the probability of event *j* from subject *i* belonging to category *k*. *β*_0_ denotes the model bias and *β_1_* is the fixed effect coefficient of variable *x*. *b_i_* is the random-effect intercept for each subject *i* which accounts for behavioral and physiological variability. $\epsilon_{ijk}$ is the model fit error.

Beta coefficient *β_1_* is the main product of this single variable association strength analysis. Term *p*/(1−*p*) is known as *odds*, hence the obtained beta coefficient can be interpreted as follows: increase in predictor variable x by 1 leads to an increase by *β_1_* in the logarithm of odds, that the observed event belongs to category *k*. As explained before, not all predictor variables were available for each observed event, hence the single variable analysis allowed to employ more data. The beta coefficients obtained separately for each predictor from **Table 1** are given in the left half of **Table 2**, with z-statistic from Wald test in parentheses.

**Table 2:** Regression analysis.

Hypoglycemia types	Separate simple regressions			Multiple regression, N=647		
	Physical activity	Carbohydrate intake	Medication intake	Physical activity	Carbohydrate intake	Medication intake
age	0.00700 (0.44)	-0.00325 (-0.26)	-0.00490 (-0.21)	0.0206* (2.05)	-0.0126 (-1.27)	-0.0456** (-3.27)
gender	0.501 (1.29)	0.311 (1.01)	-0.768 (-1.29)	0.579** (2.63)	0.353 (1.60)	-1.263*** (-3.64)
time_of_day	-2.073*** (-5.74)	1.483*** (4.35)	0.601 (1.19)	–	–	–
cos tod	-0.120 (-1.06)	-0.201 (-1.77)	0.0477 (0.29)	-0.336** (-3.17)	0.0497 (0.46)	-0.259 (-1.36)
sin_tod	1.197*** (8.50)	-0.681*** (-5.48)	-0.211 (-1.11)	0.711*** (6.36)	-0.365*** (-3.49)	-0.180 (-1.10)
wake_up_offset	-0.0200 (-0.34)	-0.00736 (-0.13)	0.00975 (0.12)	0.0955 (1.72)	-0.0679 (-1.23)	-0.0839 (-1.00)
is_bday	0.00571 (0.04)	-0.147 (-0.89)	0.568* (2.16)	0.160 (1.21)	-0.316* (-2.36)	0.544* (2.53)
bgi_mean	0.0374 (0.86)	-0.0499 (-1.12)	0.0669 (1.07)	–	–	–
bgi_std	-0.0285 (-0.45)	-0.0171 (-0.27)	0.0801 (0.90)	–	–	–
bgi_range	-0.00831 (-0.36)	-0.00627 (-0.28)	0.0237 (0.72)	–	–	–
bgi_min	0.333 (1.83)	-0.286 (-1.61)	-0.00847 (-0.03)	-0.185 (-1.21)	0.149 (0.97)	0.152 (0.76)
bgi_max	-0.00303 (-0.13)	-0.0114 (-0.49)	0.0245 (0.73)	-0.0870* (-2.27)	0.0121 (0.36)	0.0219 (0.43)
bgi_slope	-0.0854 (-0.47)	0.0179 (0.10)	-0.144 (-0.55)	-0.603* (-2.13)	-0.0511 (-0.19)	0.218 (0.58)
bgi_skew	-0.185 (-1.46)	0.00460 (0.04)	0.0328 (0.17)	-0.110 (-0.86)	0.154 (1.17)	0.0595 (0.28)
bgi_kurt	0.0191 (0.22)	-0.0195 (-0.23)	-0.109 (-0.76)	0.00563 (0.07)	-0.00837 (-0.10)	-0.0799 (-0.53)
pre_g_val	-0.0140 (-0.71)	-0.0180 (-0.90	) 0.0508 (1.82)	-0.00962 (-0.44)	-0.0224 (-1.04)	0.0742* (1.98)
pre_g_offset	-0.0700* (-2.26)	0.00715 (0.48)	-0.00318 (-0.16)	0.00355 (0.18)	-0.0158 (-0.79)	0.0217 (0.34)
pre_H_offset	-0.000870 (-0.41)	0.000833 (0.38)	-0.00694 (-1.25)	-0.00233 (-0.80)	0.00358 (1.19)	-0.00538 (-0.95)
pre_h_offset	-0.000421 (-0.51)	0.000593 (0.71)	-0.00260 (-1.63)	-0.000978 (-1.37)	0.00123 (1.76)	-0.00212 (-1.56)
pre_t_offset	-0.00873 (-0.78)	-0.00171 (-0.16)	-0.00994 (-0.71)	0.00706 (0.63)	-0.00230 (-0.21)	-0.0388* (-2.19)
post_g_val	0.0106 (0.41)	-0.0224 (-0.85)	0.0288 (0.74)	0.0299 (1.07)	-0.00300 (-0.11)	-0.0212 (-0.48)
post_g_offset	0.0517 (1.91)	-0.00882 (-0.38)	-0.0499 (-1.49)	-0.0110 (-0.28)	0.0714 (1.80)	0.0151 (0.22)
post_H_offset	0.00423 (1.20)	-0.00266 (-0.77)	-0.00243 (-0.45)	0.00579 (1.77)	-0.00534 (-1.62)	-0.00202 (-0.40)
post_h_offset	0.000534 (0.44)	-0.00212 (-1.76)	0.00206 (1.05)	0.000806 (0.85)	-0.00210* (-2.25)	0.00104 (0.65)
post_t_offset	0.0246 (1.75)	-0.00610 (-0.99)	-0.00331 (-0.42)	0.0109 (0.69)	-0.00808 (-0.54)	-0.0218 (-0.84)
pre_ins_dosage	-0.0239 (-1.81)	0.119*** (5.03)	-0.0282 (-1.64)	-0.0601** (-3.24)	0.0610*** (3.39)	0.0115 (0.44)
pre_ins offset	-0.0960*** (-3.91)	-0.00135 (-0.06)	0.0396 (1.28)	-0.0120 (-0.53)	-0.0279 (-1.28)	-0.0673 (-1.56)
post_ins_dosage	0.102*** (4.25)	-0.0195 (-1.56)	-0.0287 (-1.64)	0.0242 (1.16)	-0.0615** (-2.81)	0.0360 (1.14)
post_ins_offset	-0.0504** (-2.70)	0.0618*** (3.42)	-0.0256 (-0.93)	-0.00545 (-0.31)	0.00711 (0.39)	-0.0596 (-1.85)
pre_bas_dosage	-0.00918 (-0.50)	0.0541** (2.92)	-0.000893 (-0.04)	0.0135 (0.80)	0.0325* (2.04)	-0.000875 (-0.05)
pre_bas_offset	0.00247 (1.40)	-0.00184 (-0.94)	-0.00174 (-0.70)	-0.00554 (-0.62)	0.0110 (1.24)	-0.0790** (-3.15)
post_bas_dosage	0.0332 (1.77)	-0.0363* (-2.02)	-0.0313 (-1.40)	0.00330 (0.19)	0.0105 (0.64)	-0.0160 (-0.85)
post_bas_offset	0.00172 (0.36)	-0.000936 (-0.19)	0.00516 (0.81)	-0.00427 (-1.08)	0.00551 (1.19)	-0.0138 (-1.48)
pre_car_dosage	0.00312 (1.11)	0.000280 (0.10)	-0.0106 (-1.72)	0.00179 (0.75)	0.000499 (0.21)	-0.0179** (-3.15)
pre_car_offset	0.0104 (0.38)	-0.0476 (-1.56)	0.0231 (0.54)	-0.0219 (-0.66)	0.0112 (0.33)	0.141** (2.66)
post_car_dosage	0.00345 (1.23)	-0.00359 (-1.25)	0.00168 (0.39)	-0.00388 (-1.53)	0.00384 (1.47)	-0.00121 (-0.31)
post_car_offset	-0.0357* (-2.35)	0.0544*** (3.61)	-0.0307 (-1.23)	-0.0135 (-0.84)	0.0132 (0.81)	0.0264 (1.04)
glu_mean	-0.155 (-1.20)	0.0501 (0.44)	0.00362 (0.02)	0.186 (1.05)	-0.0494 (-0.29)	-0.601* (-2.30)
glu_std	-0.251 (-1.33)	0.0555 (0.33)	0.510 (1.60)	-0.670* (-2.19)	0.0696 (0.26)	0.558 (1.35)
glu_range	0.0275 (1.08)	-0.0176 (-0.74)	-0.00437 (-0.12)	–	–	–
glu_max	0.0309 (1.19)	-0.0195 (-0.80)	-0.00620 (-0.16)	0.00496 (0.12)	0.0349 (0.83)	0.00831 (0.13)
glu_min	0.184 (0.78)	-0.0851 (-0.38)	-0.119 (-0.36)	0.152 (0.80)	-0.158 (-0.81)	0.0789 (0.32)
glu_mean_x	-3.897 (-1.63)	1.830 (0.81)	2.500 (0.71)	-1.288 (-0.57)	0.817 (0.35)	-3.779 (-0.96)
glu_skew	0.752 (1.57)	-0.264 (-0.59)	-0.560 (-0.74)	–	–	–
glu_kurt	0.214* (2.32)	-0.106 (-1.16)	-0.246 (-1.46)	0.161 (1.21)	-0.189 (-1.42)	-0.394 (-1.67)
neg_slopes_mean	0.0820 (1.18)	-0.0899 (-1.39)	0.135 (1.12)	0.146* (2.47)	-0.166** (-2.88)	-0.0171 (-0.19)
pos_slopes_mean	0.00391 (0.03)	-0.0775 (-0.52)	0.325 (1.60)	0.157 (1.44)	-0.214 (-1.78)	0.108 (0.66)
ins_mean	-0.0227 (-0.79)	-0.0732 (-1.08)	0.0674 (1.01)	–	–	–
ins_std	-0.00337 (-0.28)	-0.132 (-1.08)	0.118 (0.63)	–	–	–
ins_range	-0.000209 (-0.28)	-0.0254 (-1.26)	0.00243 (0.08)	–	–	–
ins_max	-0.000214 (-0.26)	-0.0215 (-1.08)	0.00247 (0.08)	0.0189 (1.27)	-0.0277 (-1.78)	0.0238 (0.85)
ins_min	-0.719* (-2.35)	0.682* (2.39)	-0.0402 (-0.07)	–	–	–
ins_mean_x	-1.020 (-0.54)	5.927** (2.87)	-6.185* (-2.31)	-1.823 (-1.09)	1.424 (0.83)	2.377 (0.91)
ins_skew	-0.0990 (-0.71)	-0.0580 (-0.50)	0.192 (1.41)	-0.241 (-0.77)	0.274 (0.88)	-0.465 (-0.86)
ins_kurt	-0.0111 (-0.74)	-0.0179 (-0.57)	0.0241 (1.37)	-0.00904 (-0.16)	0.0338 (0.62)	-0.00317 (-0.03)
bas_mean	-0.0225 (-0.71)	-0.0126 (-0.48)	-0.00698 (-0.15)	–	–	–
bas_std	-0.132* (-2.05)	0.0136 (0.25)	0.241* (2.37)	–	–	–
bas_range	-0.00119(-0.05)	-0.0353 (-1.74)	0.0709* (2.25)	–	–	–
bas_max	-0.00185 (-0.09)	-0.0236 (-1.29)	0.0443 (1.37)	-0.0113 (-0.84)	0.0104 (0.72)	-0.0265 (-1.37)
bas_min	-0.00108 (-0.04)	0.0103 (0.43)	-0.0360 (-0.93)	–	–	–
bas_mean_x	-0.109 (-0.23)	-0.655 (-1.64)	0.821 (1.28)	0.123 (0.45)	-0.549* (-1.96)	-0.0573 (-0.14)
bas_skew	-0.0626 (-0.98)	-0.00487 (-0.07)	0.155 (1.49)	0.0813 (0.93)	-0.130 (-1.41)	0.147 (1.10)
bas_kurt	0.0140 (1.73)	-0.0144 (-1.41)	-0.00747 (-0.64)	0.0160 (1.57)	-0.0221 (-1.93)	0.0178 (1.30)
car_mean	-0.00644 (-0.61)	0.00289 (0.35)	0.000189 (0.01)	–	–	–
car_std	0.0120 (0.74)	-0.0112 (-0.71)	0.0145 (0.48)	0.0240 (1.03)	-0.0615* (-2.34)	0.0319 (0.79)
car_range	0.00377 (1.13)	-0.00157 (-0.52)	-0.00407 (-0.74)	–	–	–
car_max	0.00367 (1.12)	-0.00149 (-0.51)	-0.00423 (-0.79)	0.000260 (0.05)	0.0146** (2.72)	-0.0221** (-2.87)
car_min	0.00261 (0.12)	-0.000645 (-0.03)	-0.0143 (-0.43)	–	–	–
car_mean_x	0.847 (0.48)	-1.744 (-1.06)	-0.948 (-0.35)	1.431 (0.98)	-1.678 (-1.12)	0.252 (0.10)
car_skew	0.234 (1.54)	-0.184 (-1.25)	-0.208 (-0.76)	-0.0448 (-0.22)	-0.472* (-2.23)	1.002* (2.57)
car_kurt	0.00812 (0.60)	-0.00100 (-0.08)	-0.0271 (-0.46)	0.0578 (0.83)	-0.133 (-1.27)	0.0326 (0.30)
cnc_ansnum	0.00475 (1.29)	0.00225 (0.63)	-0.00700 (-1.22)	0.00181 (0.61)	0.00726* (2.40)	-0.0130** (-2.65)
cnc_daysnum	0.0378 (0.94)	0.0144 (0.37)	-0.0236 (-0.40)	–	–	–
cnc_points_avg	-0.00234 (-0.09)	-0.0110 (-0.43)	-0.00709 (-0.19)	0.0241 (1.06)	-0.0180 (-0.79)	-0.0668 (-1.84)
cnc_points std	-0.0133 (-0.43)	-0.0510 (-1.62)	0.0443 (1.00)	–	–	–
cnc_dist_avg	-0.0653 (-1.60)	-0.0226 (-0.56)	0.0429 (0.94)	-0.0187 (-0.55)	-0.0354 (-1.02)	-0.0398 (-0.93)
cnc_dist_std	-0.0135 (-0.58)	-0.0437 (-1.82)	0.0602* (1.98)	-0.0403 (-1.57)	0.0155 (0.60)	0.0663 (1.92)
ba_iob	-0.335 (-1.26)	0.308 (1.28)	-0.411 (-0.87)	–	–	–
ba_allowed_bgv	-0.0257 (-0.63)	0.0213 (0.51)	-0.00603 (-0.11)	–	–	–
ba_food_ins	-0.0581* (-2.26)	0.102*** (3.81)	-0.0456 (-1.23)	–	–	–
ba_corr_ins	0.118 (1.72)	-0.284*** (-3.63)	0.139 (1.48)	–	–	–
ba_total_ins	-0.0937*** (-3.57)	0.105*** (3.99)	-0.00760 (-0.20)	–	–	–
ba_diff	0.402* (2.51)	-0.242 (-1.83)	0.0638 (0.34)	–	–	–
ba_carb_cu	-0.464 (-1.29)	0.452 (1.14)	0.643 (1.31)	–	–	–
ba_meal_rise	-0.134 (-1.34)	0.138 (1.42)	-0.0669 (-0.39)	–	–	–
ba_has_ex	1.663*** (5.87)	-1.374*** (-4.44)	-0.749 (-1.54)	–	–	–

z statistics in parentheses; and Significance levels: *p. * p < 0.05, ** p < 0.01, *** p < 0.001. Bold styled values are statistically significant.

When considering multiple predictors simultaneously it is first necessary to select the subset of variables leading to the best model fit. To obtain more reliable effect size estimates from multiple regression analysis only the most indicative predictors were preserved. The feature quality was tested by fitting a separate MEGLM model for each individual feature and calculating the resulting Bayesian information criterion (BIC) (19), which measures the goodness of fit. After selecting a subset for every prediction target, further filtering was done by calculating the Variance Inflation Factor (VIF) (20) for every feature, and discarding those with VIF ≥ 10, thus reducing the collinearity of features. Predictors related to Bolus Advisor settings and outputs were discarded from the multiple regression analysis to increase the sample size. Prior to the analyses the dataset was centered, but not scaled.

In case of multiple regression, equation (1) takes the following form:


(3) $$ln\left(\frac{p_{ijk}}{1-p_{ijk}}\right)=\beta_{0}+\beta_{1}x_{ij}^{1}+\beta_{2}x_{ij}^{2}+\ldots +\beta_{f}x_{ij}^{f}+b_{i}+\epsilon_{ijk}\;$$

The obtained beta coefficients from multiple regression analysis are given in the right half of **Table 1** z-statistic from Wald test in parentheses. The different levels of statistical significance are marked by asterisk (* for p< 0.05, ** for p< 0.01, *** for p< 0.001). The statistical significance was defined as p< 0.05. The further interpretation of the obtained beta coefficients is given in the Results section. Data preparation was conducted in Python and statistical models were fit using STATA software.

### 2.4 Classification analysis

The aim of this analysis is to design machine learning models for automatic hypoglycemia classification and assess their performance. This system aims to automatically select one of the three categories of hypoglycemia causes - physical activity, food intake, medication intake (see **Figure 2**). The practical motivation is to design a decision support system, which would retrospectively analyze patient self-care data and highlight the possible problems in self-care to the clinician.

Two classification scenarios were investigated: binary one-vs-rest classification done separately for each class and exclusive 3-class classification. This analysis has utilized the same dataset as in multiple regression, with the Bolus Advisor features excluded to maximize the sample size.

**Figure 4:**
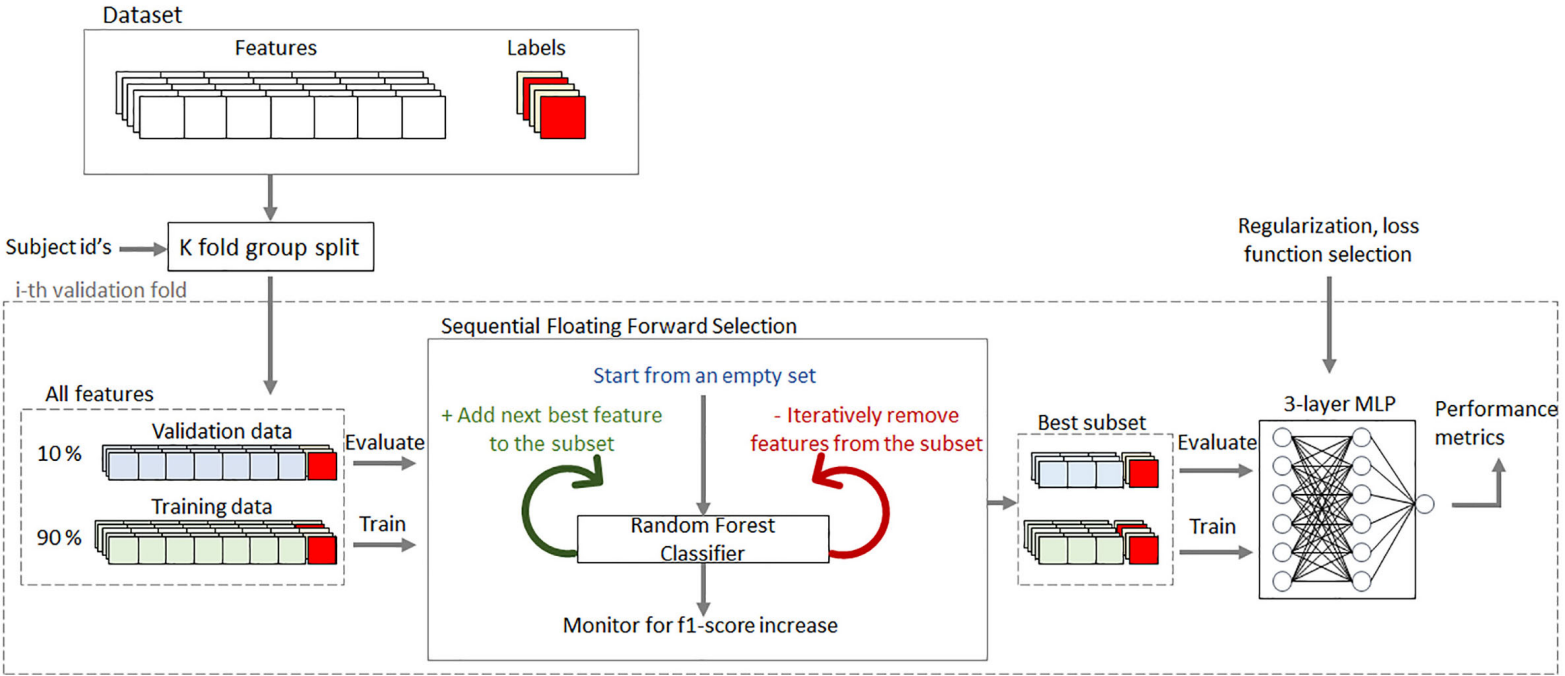
Feature selection and classifier evaluation sequence.

The analysis sequence of all investigated scenarios is illustrated in **Figure 4**. It follows the conventional supervised learning scheme consisting of feature extraction the acquired data which were represented as explained in section 2.1, feature subset selection, classifier model training and validation (21).

First the dataset was split 10 times to obtain multiple training and validation samples for the 10-fold cross-validation (CV) procedure. During the split the data was grouped by subjects to make sure that the observations from the same subject were not simultaneously present in both training and validation sets. Within each CV fold a separate feature selection procedure was applied to obtain a subset of features yielding the best classification performance. Considering the strong collinearity in the dataset, the Sequential Forward Floating Selection (SFFS) (22) algorithm was used, as it was found to be especially efficient in such settings. This algorithm iteratively adds features to the selected subset and continuously attempts to reduce the set at every iteration, while monitoring for the classification performance increase. At each iteration the performance was measured by training and validating a separate ensemble classifier in a nested 5-fold CV procedure involving only the training sample of the outer CV fold. The nested CV was also set to respect the grouping by subjects.

The SFFS algorithm is computationally expensive, therefore Extremely Randomized Tree ensemble classifier (23) was used in the SFFS procedure, since it is computationally cheap, performs well in the presence of collinear features and requires less hyperparameter fine-tuning. The final classification performance in each CV fold was assessed by training a neural network classifier (24, 25), on the selected feature subset from the training CV sample and validating it on the same subset from the validation sample. This model, consisting of 3 fully connected layers with dropout regularization, required hyperparameter optimization, was more computationally expensive to train, but has consistently outperformed the other considered classification methods in our settings. The model output consists of a single unit in binary classifiers and 3 units with softmax activation in a multi-class model, each returning the one-vs-rest probability for the corresponding class. The dropout rate was set to 60% and the number of units in each hidden layer was set equal to the number of features in the selected subset, which was decided in a separate grid search prior to the analysis. In both, SFFS procedure and neural network training steps, the observations were weighted to account for the class disbalance in the dataset. Weights for observations of class i were calculated as $w_{i}=N_{obs}/\left(N_{cl}*N_{obs}i\right)$ , where *N_obs_* is the dataset size, *N_cl_* is the number of classes and *N_obsi_* is the number of samples of class i.

Considering the strong feature collinearity, the same analysis was also conducted on predictors obtained from principal component analysis (PCA) (26) applied to the design matrix of all considered features. In this scenario the SFFS has selected generally smaller sets of orthogonal components with higher individual classification power.

The presented classification analysis has been conducted in Python using scikit-learn (27) and tensor flow (28) libraries.

#### 2.4.1 Evaluation metrics

Due to this class disbalance in each classification scenario, the simple categorical accuracy did not adequately describe classification performance, therefore Matthews correlation coefficient (MCC) (29), precision, recall and F1-score, which is the harmonic mean of the latter two, were the chosen performance metrics. The SFFS procedure aimed to maximize the mean F1-score of the classifier cross-validation. And by changing the optimization target between recall, precision and F1-score in the neural network training step it was possible to obtain different classification performance profiles, potentially suitable in the various practical settings, for example, when a certain class recall is favored over classification precision or vice-versa. By definition the chosen performance metrics are calculated for individual classes, so in the multi-class classification analysis the class-average F1-score, recall and precision were used as neural network optimization targets.

## 3 Results

### 3.1 Statistical analysis

This analysis has highlighted a number of interpretable predictors of different hypoglycemia types. For example, as the statistical significance of multiple regression predictors suggests, episodes related to physical activity generally happened during daytime, were associated with lower QA insulin intake prior to event, were more common in men, in subjects with lower glycemic variability and slower BG fall in general. Episodes related to food intake had a higher probability to occur in mornings, evenings and on weekends, were associated with higher relative QA insulin dosages before the event and lower dosages after, were more commonly observed in subjects with slower general BG decline, frequent changes in basal insulin dosages and more frequent consumption of large amounts of food. Hypoglycemic episodes related to mistakes in medication dosage selection were more common in women, subjects with lower average BG levels, more frequent consumption of small amounts of food and less activity in the GlucoChallenge quiz; occurred more frequently during the business days and generally later after the food intake.

Simple regression scenarios show that the features extracted from Bolus Advisor settings, tags and most recent advice prior to the episodes are strong predictors of activity- and food-related hypoglycemia classes, however they were available in only 328 cases.

### 3.2 Classification analysis

The results of classification analysis are detailed in **Table 3**. The SFFS procedure aimed to maximize the mean F1-score of the classifier cross-validation. And by changing the optimization target between recall, precision and F1-score in the neural network training step it was possible to obtain different classification performance profiles, potentially suitable in the various practical settings, for example, when a certain class recall is favored over classification precision or vice-versa. By definition the chosen performance metrics are calculated for individual classes, so in the multi-class classification analysis the class-average F1-score, recall and precision were used as neural network optimization targets. The classification analysis was also conducted on predictors obtained from PCA applied to the design matrix of all considered features. The results of the described CV analyses are combined in **Table 3**, which contains the average performance metrics from CV with standard deviations.

**Table 3:** Classification analysis results.

	Binary One-vs-Rest classification						
	Raw features				PC features		
NN Objective	Result metrics	Physical activity	Food intake	Medication intake	Physical activity	Food intake	Medication intake
F1-score	F1-score	0.65 ± 0.11	0.49 ± 0.12	0.39 ± 0.23	0.67 ± 0.13	0.49 ± 0.13	0.30 ± 0.12
	Recall	0.84 ± 0.08	0.73 ± 0.21	0.63 ± 0.23	0.87 ± 0.06	0.79 ± 0.26	0.61 ± 0.25
	Precision	0.55 ± 0.16	0.39 ± 0.12	0.33 ± 0.28	0.56 ± 0.16	0.39 ± 0.10	0.22 ± 0.11
	MCC	0.33 ± 0.05	0.19 ± 0.06	0.23 ± 0.25	0.34 ± 0.10	0.16 ± 0.07	0.12 ± 0.14
Recall	F1-score	0.60 ± 0.14	0.47 ± 0.14	0.27 ± 0.17	0.63 ± 0.15	0.48 ± 0.13	0.24 ± 0.16
	Recall	0.96 ± 0.06	0.83 ± 0.16	0.75 ± 0.18	0.97 ± 0.04	0.91 ± 0.13	0.88 ± 0.13
	Precision	0.46 ± 0.19	0.35 ± 0.15	0.18 ± 0.14	0.48 ± 0.17	0.33 ± 0.12	0.16 ± 0.13
	MCC	0.15 ± 0.15	0.12 ± 0.11	0.05 ± 0.14	0.25 ± 0.08	0.09 ± 0.04	0.05 ± 0.09
Precision	F1-score	0.46 ± 0.17	0.32 ± 0.13	0.28 ± 0.18	0.39 ± 0.16	0.23 ± 0.07	0.25 ± 0.06
	Recall	0.46 ± 0.24	0.34 ± 0.26	0.36 ± 0.20	0.29 ± 0.16	0.16 ± 0.07	0.45 ± 0.29
	Precision	0.67 ± 0.22	0.60 ± 0.26	0.33 ± 0.30	0.71 ± 0.18	0.57 ± 0.24	0.33 ± 0.31
	MCC	0.24 ± 0.06	0.15 ± 0.11	0.16 ± 0.22	0.25 ± 0.12	0.15 ± 0.07	0.19 ± 0.07
NN Objective	Raw features				PC features	Food intake	Medication intake
	Result metrics	Physical activity	Food intake	Medication intake	Physical activity		
F1-score	F1-score	0.51 ± 0.25	0.43 ± 0.16	0.06 ± 0.14	0.44 ± 0.25	0.19 ± 0.21	0.09 ± 0.11
	Recall	0.54 ± 0.30	0.56 ± 0.29	0.05 ± 0.11	0.61 ± 0.43	0.38 ± 0.43	0.06 ± 0.08
	Precision	0.51 ± 0.24	0.40 ± 0.13	0.08 ± 0.19	0.47 ± 0.23	0.20 ± 0.20	0.29 ± 0.38
	MCC		0.13 ± 0.11			0.06 ± 0.06	
Recall	F1-score	0.52 ± 0.26	0.34 ± 0.24	0.09 ± 0.15	0.46 ± 0.19	0.32 ± 0.16	0.06 ± 0.08
	Recall	0.59 ± 0.3	4 0.48 ± 0.38	0.08 ± 0.13	0.51 ± 0.30	0.50 ± 0.31	0.06 ± 0.10
	Precision	0.51 ± 0.24	0.33 ± 0.20	0.11 ± 0.19	0.54 ± 0.15	0.33 ± 0.24	0.22 ± 0.34
	MCC		0.11 ± 0.14			0.08 ± 0.09	
Precision	F1-score	0.50 ± 0.20	0.41 ± 0.15	0.01 ± 0.02	0.53 ± 0.22	0.30 ± 0.23	0.07 ± 0.11
	Recall	0.55 ± 0.33	0.56 ± 0.30	0.01 ± 0.01	0.62 ± 0.28	0.41 ± 0.32	0.05 ± 0.09
	Precision	0.57 ± 0.11	0.41 ± 0.20	0.14 ± 0.35	0.48 ± 0.23	0.25 ± 0.19	0.14 ± 0.24
	MCC		0.12 ± 0.09			0.06 ± 0.06	

MCC, Matthews correlation coefficient.

According to the results in **Table 3**, in all classification scenarios the accuracy metrics were proportionate to the corresponding class support. The highest F1-score for the most common class of activity-related episode was 0.67 ± 0.13 and 0.51 ± 0.25 in binary and multiclass classification scenarios respectively; 0.49 ± 0.12 and 0.43 ± 0.16 for a less represented class of food intake related episodes; 0.39 ± 0.23 and 0.6 ± 0.14 for the least common class of episodes related to medication intake. Despite the sampling weighting, detection of insulin-related episodes was rarely possible in the multiclass classification scenario.

The different performance profiles were successfully produced by changing the ANN training objective between F1-score, recall and precision. It can be speculated that for practical implementation in a decision support system, the F1-score and precision are more suitable choices of the target performance metric.

PCA transformation of the raw handcrafted features was especially beneficial in one-vs-rest classification scenario. Besides, it has significantly sped up the exhaustive SFFS procedure. Thus, the application of other PCA variations and dimensionality reduction methods is also of interest.

## 4 Discussion

The conducted feasibility study has outlined a number of concerns valuable in the design of the decision support system for automatic hypoglycemia reason classification. Firstly, our data acquisition has characterized the incidence distribution of the various hypoglycemia reasons with more than 45% of the collected cases being related to physical activity.

Secondly, the conducted statistical and classification analyses have highlighted many interpretable predictors of the various hypoglycemia types. These predictors were designed manually to describe the subject self-care around the observed episodes, as well as their general self-care habits. Although a relatively large number of predictors was considered, the further search for optimal data features based on clinical domain knowledge can potentially improve the performance of the desirable reasoning system. Besides that, deep learning (24) could be applied to this problem to automatically design features directly from the raw collected data, however such features would not be human-interpretable.

Thirdly, the classification analysis has characterized the potential performance and limitations of the proposed system in the current settings, where reasoning relies on data manually entered by subjects. As it currently stands, practical application of automatic hypoglycemia cause inference is questionable, due to the lack of self-care and physiological data. Statistical analysis results and manual visual assessment of subject self-care records show that food intake data is often omitted from personal diaries, especially snacks and food intake to correct hypoglycemia. This hinders the understanding and modeling of BG dynamics, and consequently, the classification performance.

While the objective unobtrusive measurement of food intake is problematic, continuous physical activity tracking in users *via* the commercially available wearable sensors is viable. Even though it was not measured in our experimental settings, classification analysis results show that activity-related episodes were the easiest to detect (**Table 3**), partially due to the amount of relevant cases in the dataset. It can be speculated that the introduction of activity tracking in subjects would significantly increase the rate and accuracy of this hypoglycemia class detection, which in turn would also reduce the uncertainty about other classes. Therefore it can be stated that the addition of physical activity tracking in Glucollector and other BG decision support systems is the key necessary element to make this type of reasoning feasible and beneficial in clinical practice.

## Data availability statement

The datasets presented in this article are not readily available because the data is only available to the researchers of DAFNEplus. Requests to access the datasets should be directed to ME, m.eissa@sheffield.ac.uk.

## Ethics statement

The studies involving human participants were reviewed and approved by National Health Service Research Ethics Committee (REC ref: 16/WS/0230 and 18/SW/0100). The patients/participants provided their written informed consent to participate in this study.

## Author contributions

ME, ZH, and TG designed the visual interfaces and collected the data. AZ, ME, and ZH analyzed the data and AZ performed the experiment. JE and MB verified the outcomes. AZ and ME prepared the manuscript with the help and review of all authors. All authors contributed to the article and approved the submitted version.
