# Corrigendum: Automatic inference of hypoglycemia causes in type 1 diabetes: a feasibility study

**DOI:** 10.3389/fcdhc.2023.1227105

**Published:** 2023-06-07

**Authors:** Aleksandr Zaitcev, Mohammad R. Eissa, Zheng Hui, Tim Good, Jackie Elliott, Mohammed Benaissa

**Affiliations:** ^1^ Department of Electronic and Electrical Engineering, University of Sheffield, Sheffield, United Kingdom; ^2^ Department of Oncology and Metabolism, University of Sheffield, Sheffield, United Kingdom; ^3^ Department of Diabetes and Endocrinology, Sheffield Teaching Hospitals NHS FT, Sheffield, United Kingdom

**Keywords:** biomedical informatics, classification algorithms, machine learning, medical expert systems, statistical analysis, hypoglycemia, exercise, physical activity

In the published article, the supporting funder, University of Sheffield Institutional Open Access Fund was erroneously omitted in the Funding statement as published. Instead of

This study is funded by the National Institute for Health Research (NIHR) [Programme Grants for Applied Research (RP-PG-0514-20013) DAFNEplus]. It should be

This study is funded by the National Institute for Health Research (NIHR) [Programme Grants for Applied Research (RP-PG-0514-20013) DAFNEplus]. This article was also supported by University of Sheffield Institutional Open Access Fund.

The authors apologize for this error and state that this does not change the scientific conclusions of the article in any way. The original article has been updated.

